# Retrospective analysis of hospital episode statistics, involuntary admissions under the Mental Health Act 1983, and number of psychiatric beds in England 1996-2006

**DOI:** 10.1136/bmj.a1837

**Published:** 2008-10-09

**Authors:** Patrick Keown, Gavin Mercer, Jan Scott

**Affiliations:** 1 East Community Mental Health Team, Molineux Street NHS Centre, Newcastle upon Tyne NE6 1SG; 2 University Department of Psychiatry, Royal Victoria Infirmary, Newcastle upon Tyne

## Abstract

**Objective** To analyse the number of voluntary and involuntary (detentions under the Mental Health Act 1983) admissions for mental disorders between 1996 and 2006 in England.

**Design** Retrospective analysis.

**Setting** England.

**Main outcome measures** Number of voluntary and involuntary admissions for mental disorders in England’s health service, number of involuntary admissions to private beds, and number of NHS beds for patients with mental disorders or learning disabilities.

**Results** Admissions for mental disorders in the NHS in England peaked in 1998 and then started to fall. Reductions in admissions were confined to patients with depression, learning disabilities, or dementia. Admissions for schizophrenic and manic disorders did not change whereas those for drug and alcohol problems increased. The number of NHS psychiatric beds decreased by 29%. The total number of involuntary admissions per annum increased by 20%, with a threefold increase in the likelihood of admission to a private facility. Patients admitted involuntarily occupied 23% of NHS psychiatric beds in 1996 but 36% in 2006.

**Conclusions** Psychiatric inpatient care changed considerably in the decade from 1996 to 2006, with more involuntary admissions to fewer NHS beds. The case mix has shifted further towards psychotic and substance misuse disorders, which has changed the milieu of inpatient wards. Increasing proportions of involuntary patients were admitted to private facilities.

## Introduction

Between 1955 and 1995 deinstitutionalisation resulted in the number of beds for mental illness and learning disability in England’s health service (NHS) decreasing from over 150 000^1^ to fewer than 55 000. Although some evidence suggests that crisis teams and services for early intervention in psychosis can reduce the number of admissions for mental disorders compared with traditional psychiatric provision,^2^ ^3^ one study^4^ found that crisis teams reduce the number of voluntary admissions but not involuntary admissions—detentions under the Mental Health Act 1983. Observers are debating whether a new era of reinstitutionalisation has begun.^5^

We examined changes in the number of psychiatric admissions in England from 1996 to 2006, explored any associations between reductions in NHS bed numbers and involuntary admission rates, and calculated the proportion of involuntary inpatients being treated in non-NHS facilities.

## Methods

We submitted a request to the NHS Information Centre for data on admissions to NHS hospitals in England from hospital episode statistics. We obtained information on admissions for all mental and behavioural disorders (codes F00-F99, international classification of diseases, 10th revision). To estimate the number of psychiatric beds we combined data on the number of available mental illness and learning disability beds from hospital activity statistics.^6^

Cross sectional data on numbers of involuntary inpatients in NHS hospital and private facilities on 31 March each year were derived from the Department of Health.^7^ We calculated the total number of involuntary admissions per annum by combining the numbers of patients detained under the Mental Health Act—civil, forensic (patients involved in criminal proceedings), and place of safety. Patients whose status changed (from place of safety to civil) were included only once, in the estimates for place of safety. The “count me in census” (the number of patients admitted for mental disorders in NHS and independent hospitals in England and Wales counted on one day each year) provided data on ethnicity of voluntary and involuntary psychiatric inpatients on 31 March 2006. We carried out statistical analyses using Pearson correlations.

## Results

Admissions to NHS hospitals for mental disorders peaked in 1998, at 214 326. Over the study period from 1996 to 2006, yearly admissions declined by 11%, from 207 050 to 183 699. The reductions were confined to three subpopulations (figure[Fig fig1]): patients with learning disabilities (admission decreased by 58%), unipolar depression (33%), or dementia (28%). Since 2003, admissions for drug and alcohol related disorders increased by 29%, whereas admissions remained relatively stable for other groups—for example, 30 639 patients were admitted for schizophreniform disorders in 1996 compared with 30 852 in 2006.

**Figure fig1:**
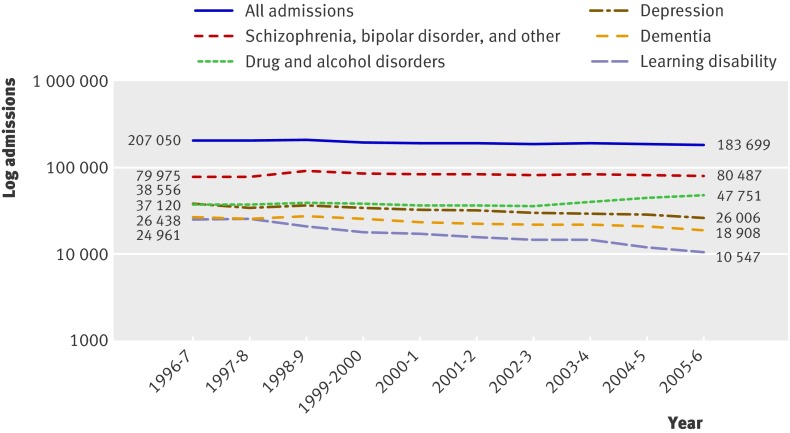
Changes in admission rates (on logarithmic scale) to NHS hospitals for different diagnostic groups of mental disorders, 1996-2006

Over the decade the total number of involuntary admissions per annum increased by 20%, from 42 844 to 51 361; the number of civil detentions increased by 13%, from 38 938 to 43 820, and place of safety detentions increased by 189%, from 2037 to 5877, whereas forensic detentions decreased by 11%, from 1873 to 1664. The table[Table tbl1] shows that on 31 March each year the number of involuntary inpatients in hospital increased by 27%, from 11 500 to 14 625, with noticeable increases in the numbers admitted to private facilities compared with NHS facilities (256% *v* 12%). The odds of an involuntary patient being admitted to hospital in a private facility increased threefold (odds ratio 3.2, 95% confidence interval 2.9 to 3.5), from 0.06 to 0.21 (700/10 800 patients *v* 2493/ 12 132).

**Table tbl1:** Number of beds for mental illness and learning disability, and number of involuntary inpatients between 1996 and 2006, in England

Variable	1996-7	1997-8	1998-9	1999-2000	2000-1	2001-2	2002-3	2003-4	2004-5	2005-6	% change	Pearson correlation*
No of NHS beds for mental disorders and learning disability	47 333	44 798	43 183	41 007	40 530	38 477	37 791	37 464	35 701	33 729	29% reduction	—
Involuntary inpatients on 31 March:												
No in NHS facilities	10 800	11 800	11 823	11 473	12 150	11 566	11 589	11 708	12 148	12 132	12% increase	r=−0.64; P=0.046
No in private facilities	700	850	1170	1382	1679	1893	1999	2292	2533	2493	256% increase	r=−0.98; P<0.001
Total No	11 500	12 700	12 993	12 855	13 829	13 459	13 588	14 000	14 681	14 625	27% increase	r=−0.94; P<0.001
% of NHS beds occupied by involuntary inpatients†	23	26	27	28	30	30	31	31	34	36	—	—

*Correlation with number of NHS beds for mental disorder and learning disability.

†Number of involuntary patients in NHS facilities on 31 March divided by number of NHS beds for mental disorders and learning disability and multiplied by 100.

As the number of psychiatric beds decreased by 29% (47 333 to 33 729), the proportion of NHS beds occupied by involuntary patients increased, from 23% to 36% (table[Table tbl1]). A negative correlation was seen between the number of NHS beds and the number of involuntary patients in private facilities (r=−0.98, P<0.001) or in NHS facilities (r=−0.64, P=0.046).

The “count me in census” on 31 March 2006 identified 30 023 patients with mental disorders in NHS hospitals and 4262 in private facilities in England; 40% of NHS inpatients compared with 58% of private inpatients were involuntary. Ethnic minority groups comprised 21% of NHS inpatients, 19% of private inpatients, and 28% of all involuntary inpatients.

## Discussion

The number of involuntary admissions for mental disorders in England per annum increased by 20% from 1996 to 2006, whereas the total number of admissions and number of NHS psychiatric beds decreased. Reductions in admissions have largely been confined to patients with depression, learning disability, or dementia. The increase in numbers of involuntary inpatients has been most noticeable in private facilities: in 1996-7 involuntary patients were 15 times more likely to be in an NHS facility than a private facility, but by 2006 only five times more likely.

Limitations of the study are that findings only apply to England and most data sources warned of difficulties in comparing year on year estimates—a problem likely to be amplified by our comparisons across databases. That the data derived from disparate sources indicate similar trends, however, suggests that we are observing real changes in inpatient activity.

These findings support previous studies showing an increase in number of involuntary admissions^8^ ^9^ against a background of reductions in numbers of NHS beds. Psychotic and affective disorders account for over 50% of all NHS occupied psychiatric bed days in England; but changes in acute admissions were primarily accounted for by reductions in voluntary admissions for depression, a group with the shortest length of stay (data available on request). Although this may reflect the redirection of patients with depression to crisis teams, the impact on acute inpatient wards has been dramatic, with shifts in case mix (toward psychoses and drug and alcohol misuse) and extended lengths of stay (involuntary inpatients).

The increase in number of involuntary admissions to private facilities is noteworthy as the NHS purchases about 80% of private psychiatric provision. One of the largest growth areas has been in the provision of private medium secure beds even though NHS forensic facilities have expanded. The decrease in forensic involuntary admissions is therefore surprising. A question for further research is whether the changes identified in this study have applied across demographic and diagnostic groups and NHS regions for the duration of the Mental Health Act 1983.

### Conclusions

Psychiatric inpatient care changed considerably from 1996 to 2006, with more involuntary patients admitted to fewer NHS beds and increasing proportions of involuntary patients admitted to private facilities. The decrease in acute general adult admissions has been confined to voluntary patients with depression. The inpatient case mix has shifted further towards psychotic and substance misuse disorders, which has changed the milieu on inpatient psychiatric wards.

**What is already known on this topic**

Rates of involuntary admissions under the Mental Health Act 1983 increased in the 1980s and ’90s
The number of psychiatric beds in the NHS has been decreasing since the 1950s

**What this study adds**

The number of patients admitted to the NHS with mental disorders peaked in 1998 and is now decreasing
The number of psychiatric beds in the NHS has continued to fall and rates of involuntary admissions have continued to rise
A greater proportion of psychiatric inpatients are now involuntary and are being treated in private facilities

## Notes

Reuse of information from the “count me in census” and other datasets was covered by the public sector information licence held by Newcastle University.

Ethical approval: Not required.

Provenance and peer review: Not commissioned; externally peer reviewed.

## Notes

**Cite this as: ***BMJ* 2008;337:a1837
